# Couples' daily self-regulation: The Health Action Process Approach at the dyadic level

**DOI:** 10.1371/journal.pone.0205887

**Published:** 2018-10-29

**Authors:** Corina Berli, Janina Lüscher, Aleksandra Luszczynska, Ralf Schwarzer, Urte Scholz

**Affiliations:** 1 Department of Psychology, University of Zurich, Zurich, Switzerland; 2 Trauma, Health, & Hazards Center, University of Colorado, Colorado Springs (CO), United States of America; 3 Psychology Department, SWPS University of Social Sciences and Humanities, Wroclaw, Poland; 4 Department of Education and Psychology, Freie Universität Berlin, Berlin, Germany; 5 University Research Priority Program “Dynamics of Healthy Aging”, University of Zurich, Zurich, Switzerland; Qazvin University of Medical Sciences, ISLAMIC REPUBLIC OF IRAN

## Abstract

**Objective:**

Regulating health behavior change often occurs in a dyadic context of romantic relationships. Dyadic approaches to standard health behavior change models are, however, barely considered. We investigated volitional processes of the Health Action Process Approach model for two health behaviors within a dyadic context of romantic couples. Specifically, we tested whether day-to-day volitional self-regulation predicted one’s own and one’s partner’s cigarettes smoked (Study 1) and physical activity (Study 2).

**Methods:**

In two dyadic intensive longitudinal studies (Study 1: 83 dual-smoker couples intending to jointly quit smoking; Study 2: 61 overweight couples intending to become physically active), heterosexual partners independently reported on intention, self-efficacy, action planning, and action control in end-of-day diaries. In Study 1, daily number of cigarettes smoked was assessed via self-report. In Study 2, daily moderate-to-vigorous physical activity (MVPA) was assessed objectively via accelerometers. In both studies, dyadic cross-lagged intensive longitudinal analyses based on the Actor-Partner Interdependence Model were applied.

**Results:**

Across both studies, individual’s own volitional self-regulation positively predicted one’s own health behavior (less cigarettes smoked and more MVPA). One’s partner’s action control and intention also positively predicted one’s own health behavior. A marginal partner effect for self-efficacy was found in the context of smoking only.

**Conclusions:**

Behavioral self-regulation is not only relevant for individuals themselves, but some volitional processes may spill over to their partners. This highlights the need to specify couple-level processes involved in health behavior change, and to consider a social context of self-regulation.

## Introduction

Despite best intentions, performing a health behavior in daily life requires constant self-regulatory efforts [1]. The Health Action Process Approach model (HAPA; [1]) assumes that volitional processes (e.g., self-efficacy, action planning, action control) are of particular importance for effective health behavior change in motivated individuals. However, the HAPA focuses primarily on individual regulation capacities as determinants for successful health behavior change, and does not specifically address the role of the social context. This is despite the fact that daily life often unfolds within dyadic relationships [2]. Studying romantic couples, for example, allows to examine individual processes of health behavior change models at the dyadic level. The present study thus investigates romantic couples’ self-regulation in daily life, testing whether volitional HAPA processes do not only predict one’s own, but also one’s partner’s health behavior. Study 1 examined dual-smoker couples intending to quit smoking, whereas Study 2 examined overweight couples intending to become more physically active.

### Volitional self-regulation in health behavior change

The HAPA provides a theoretical framework for identifying important processes for health behavior change [1]. It explicitly distinguishes between a motivational phase leading to the formation of an intention, and a volitional phase leading to the actual health behavior and its maintenance. Once an intention has been set, it is assumed that self-efficacy, action planning, and action control are important for initiation and maintenance of the intended behavior. *Self-efficacy* refers to believing in one’s capability to perform a desired action by one’s own resources [3]. *Action planning* refers to generating concrete plans about when, where, and how to perform a health behavior [4]. In contrast to these prospective self-regulatory strategies (i.e., intention, self-efficacy, action planning), *action control* is a self-regulatory strategy concurrent to ongoing behavior, comprising three subfacets: awareness of standards (i.e., being aware of one’s intentions), self-monitoring (i.e., observing one’s behavior in order to evaluate whether it corresponds with one’s intentions), and self-regulatory effort (i.e., applying means to reduce discrepancies between one’s behavior and intentions) [5].

Overall, the HAPA has demonstrated applicability across a variety of health behaviors [1], including the context of smoking cessation (e.g., [6]) and physical activity [7–9]. Most evidence comes from analyses at the between-person level (i.e., detecting differences across persons), but recent studies provided evidence that the HAPA-assumed associations between volitional processes and health behavior hold at the within-person level (i.e., detecting differences across occasions within individuals). For example, self-efficacy, action planning, and action control predicted a lower number of cigarettes smoked at the daily level before and after a self-set quit date [10]. Similarly, volitional predictors intention, self-efficacy, and action control were found to be associated with higher levels of university students’ physical activity across nine biweekly assessments [9]. Maher and Conroy [11] demonstrated that planning to reduce sedentary behavior was associated with less hours of sedentary behavior within individuals. Bierbauer et al. [12] confirmed action control as the sole significant predictor of physical activity at the within-person level in a sample of older adults.

### A dyadic perspective on health behavior change

The focus of a vast majority of health behavior change models, such as the HAPA, is mainly on intra-individual processes. However, individuals usually try to change their health behavior while being embedded in a social environment of romantic partners, family, or friends. According to the United Nations [13], approximately 80% of persons aged 45 to 49 years lived in a committed relationship during some part of their adult lifespan.

Empirical evidence shows that close relationships impact physical health profoundly (e.g., [14, 15]). Spouses have similar health status, which is possibly due to shared common lifestyles [16]. There is consistent evidence that romantic partners are highly concordant in their health behaviors [17, 18], and that making a health behavior change is closely linked within couples [17, 19]. In line with this, the Transactive Goal Dynamics theory (TGD; [20]) conceptualizes two interdependent individuals as one single self-regulating system. According to the TGD, both individuals in a TGD system can pursue goals that are self-oriented, partner-oriented or couple-oriented, and each individuals’ goal pursuits are strongly interrelated. However, specific dyadic processes involved in driving behavior change still need to be clarified and empirically tested. For example, it is unclear if the interdependence in health behavior among romantic couples is due to interdependent self-regulation processes? To the best of our knowledge, this has not yet been empirically tested. Moreover, can we assume that in the context of romantic couples, one partner’s individual effort to regulate health behavior change can contribute (or ‘spillover’) to the other partner’s health behavior change? A dyadic version of a health behavior change model, accounting for both partners’ individual perspectives on self-regulation processes in health behavior change would allow to understand these processes more fully.

So far, research suggested that spouses’ views of patient’s efficacy is highly predictive for outcomes in congestive heart failure patients (e.g., [21]). Only one study has tested spillover effects of individual processes in health behavior change: Howland et al. [22] applied the Theory of Planned Behavior (TPB; [23]) in a dyadic context, and found that partner’s perceived behavioral control was significantly associated with one’s own intention to exercise over and above the effect of one’s own perceived behavioral control (partner effect). The remaining TPB predictors (attitudes and subjective norms) did not yield any partner effects. The TPB considers motivational processes of health behavior change, predicting intention to perform a behavior, but not necessarily the post-intentional, volitional processes. To date, no study applied such a dyadic approach with regard to volitional processes of health behavior change. Moreover, Howland et al. [22] used a cross-sectional design to test a between-person perspective. To better understand the fine-grained processes underlying health behavior change, applying a within-person perspective is essential. The present study aims to address these issues, using dyadic intensive longitudinal data from two studies.

### Aims of the present study

**Fig 1:**
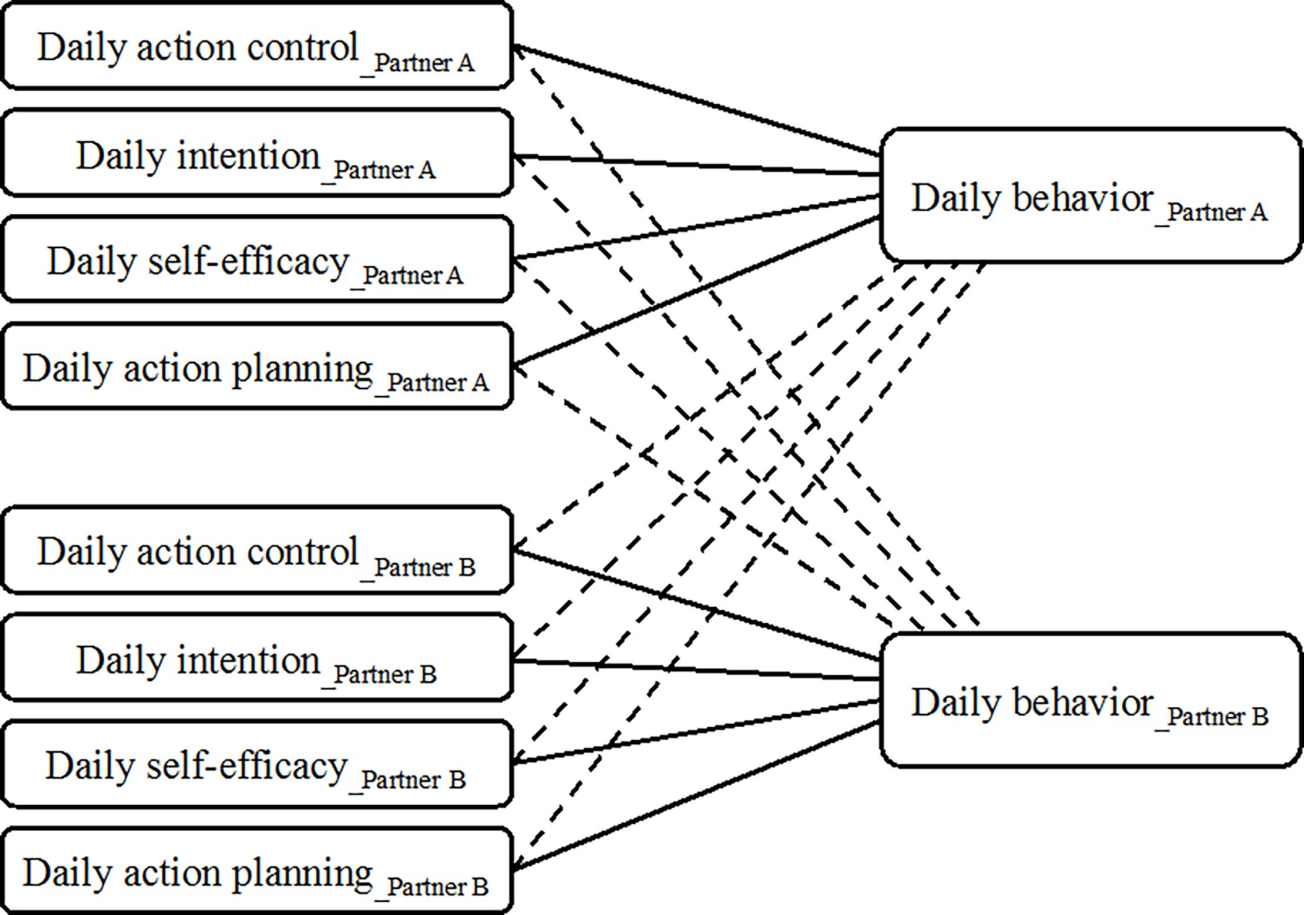
Hypothesized dyadic version of the Health Action Process Approach in couples where one’s own volitional processes predict better own health behavior (actor effects represented with solid lines) and one’s partner’s volitional processes predict better own health behavior (partner effects represented with dashed lines). *Note*: The conceptual model is based on the Actor-Partner Interdependence Model (APIM; [24]) depicting two dyad members, Partner A and Partner B, with predictors and outcome for each member. The dyadic models were calculated separately for each volitional predictor.

We aimed to examine the role of volitional HAPA processes for daily health behaviors in a dyadic context of romantic couples: Dual-smoker couples intending to jointly quit smoking (Study 1) and couples with obesity, intending to become physically active (Study 2). First, we descriptively examined the concordance of romantic partners in self-regulating their health behaviors in daily life. Second, we examined how volitional processes relate to health behaviors in daily life within a dyadic version of the HAPA, applying the Actor-Partner Interdependence Model (APIM; [24]). We focused on variations across days within individuals and dyads. *Fig 1* shows the conceptual model. Specifically, we hypothesized that (1) one’s own action control as a concurrent self-regulation strategy would be associated with better health behavior that same day, and (2) that one’s own intention, self-efficacy, and action planning reported prospectively for the next day would be associated with better health behavior the next day (actor effects). Moreover, we hypothesized that (3) volitional HAPA processes would not only predict better one’s own health behavior but also better one’s partner’s health behavior (partner effects) the same day or the next day.

## Methods

### Design

Study 1 was part of a larger prospective longitudinal project with correlational design investigating individual self-regulation and dyadic exchanges in dual-smoker couples around a joint self-set quit date. For more details please see Lüscher et al. [25]. The project was funded by the Swiss National Science Foundation (PP00P1_133632/1) and approved by the Internal Review Board of the University of Bern, Switzerland (2011-11-14409). Participating couples were invited to the lab where they provided written informed consent and completed an online questionnaire including socio-demographic data. They were asked to jointly set on a quit date, but did not receive any type of intervention. Across 32 consecutive days around the joint self-set quit date, couples reported on daily experiences and their smoking in end-of-day diaries using smartphones provided to them. Couples were instructed to fill in the daily survey each night within one hour of going to bed, separately from each other, starting 10 days before the quit date and for 21 days afterwards. They were asked not to discuss their answers with their partners. To capture effects of daily volitional HAPA processes during the joint self-set quit attempt, the present analyses focus on the quit date day and the following 21 days. At one month after the joint quit date participating couples returned to the laboratory for a follow-up and completed biochemical verification of smoking status with a carbon monoxide test of expelled air [26]. At baseline, 85 couples participated in the study. As two couples dropped out before the joint self-set quit date, 83 couples served as sample for the present analyses.

Study 2 was part of an intervention study to promote daily physical activity in inactive couples with overweight, intending to become physically active (‘DYACTIC’; for detailled information please see Scholz & Berli [27]). The project was funded by the Swiss National Science Foundation (PP00P1_133632/1) and approved by the Internal Review Board of the University of Bern, Switzerland (2011-12-36206). The present study is based solely on data provided by couples participating in the control condition (receiving no self-regulatory intervention, but information on physical activity only). Of the 62 couples randomized to the control condition, 61 couples completed baseline assessments and served as sample for the present analyses. More information on recruitment and sampling procedures as well as the results on the intervention effect are reported in detail elsewhere [28]. Similar to the procedure in Study 1, participating couples were invited to the lab where they provided written informed consent and completed an online questionnaire. They received an information leaflet with physical activity recommendations for adults based on guidelines by the Swiss Federal office of Sports at the time of the study (engaging in 30 min or more of at least moderate activity every day, performed in bouts of at least 10 minutes; [29]). For a diary period of 28 consecutive days (starting the day after baseline) couples independently filled in an electronic end-of-day diary on a provided smartphone within one hour of going to bed. As in Study 1, they were asked not to discuss their answers with their partners. Accelerometers were handed out for the assessment of physical activity in both partners across the 28 days. After this period, couples returned to the lab to return the devices and complete a follow-up assessment.

In both studies the participating couples were compensated with CHF 100 (= 97 USD) for study participation, independent of the number of completed diaries.

### Participants

Participants were recruited via newspapers, web pages, public advertising, bulletins, and a market research institution. In Study 1, participants were 83 heterosexual adult dual-smoker couples living in a committed relationship for at least one year (*M* = 12.68 years, *SD* = 12.79) and cohabitating for at least six months (*M* = 11.08 years, *SD* = 12.95). Both partners smoked at least one cigarette daily and reported intention to quit smoking on a joint self-set quit date during the study. Mean number of cigarettes smoked was 14.33 for women (*SD* = 6.53, range: 2–30) and 17.45 for men (*SD* = 8.85, range: 2–60) at the baseline. On average women were 38.51 years old (*SD* = 14.57, range: 19–68) and men were 40.72 years old (*SD* = 14.51, range: 20–71).

In Study 2, participants were 61 heterosexual adult couples living in a committed relationship for at least one year (*M* = 17.97 years, *SD* = 14.31) and cohabitating for at least six months (*M* = 15.90 years, *SD* = 14.52). Both partners were overweight or obese (Body Mass Index [BMI] ≥ 25 kg/m^2^); the average BMI was 30.02 (*SD* = 4.25, range: 25–46) among women and 30.77 (*SD* = 4.09, range: 25–49) among men. Both partners did not meet physical activity recommendations [29], but intended to increase their physical activity levels. On average women were 43.39 years old (*SD* = 13.67, range: 22–68); men were 45.07 years old (*SD* = 13.92, range: 22–72). In both studies, exclusion criteria were insufficient knowledge of the German language, pregnancy, and enrollment into a professional smoking cessation (Study 1) or weight loss (Study 2) program.

### Measures

The end-of-day-diaries contained (single item) measures of volitional HAPA variables adapted from scales by Scholz et al. [6], with a response format of 1 (today not at all true) to 6 (today completely true). Overall, the participating couples showed high diary completion rates (Study 1: *n* = 3,031 [83.0%] of 3,652 possible diary days; Study 2: *n* = 3,044 [92.4%] of 3,294 possible diary days). Table 1 gives an overview of the items of volitional HAPA variables and descriptive statistics of the variables of interest for both studies. As can be seen, in Study 1 only one of the three subfacets of action control (self-monitoring) was assessed. The outcome measures were assessed as follows:

**Table 1:** Descriptive statistics of variables of interest.

	*Item*	*N* ^*a*^	*n*	*M*	*SD*_*W*_	*R*_*W*:_ *M(SD)*	*R*_*w*_: *Min*, *Max*
*Study 1*: *Smoking*							
1. Daily cigarettes smoked		83 (166)	3026	4.27	2.54	.37(0.38)	-0.50, 1.00
2. Daily Action Control	Today I constantly monitored whether I acted the way I intended to in terms of my smoking. *(self-monitoring)*	83 (166)	3031	4.00	1.30	.12(0.30)	-0.69, 0.89
3. Daily Intentions	I have the intention to refrain from smoking tomorrow.	83 (166)	3031	4.88	0.87	.19(0.35)	-0.58, 0.76
4. Daily Self-Efficacy	I am confident that I can refrain from smoking tomorrow even if it is difficult.	83 (166)	3031	4.62	1.00	.16(0.38)	-0.60, 1.00
5. Daily Action Planning	I have made a detailed plan for tomorrow as to how I achieve not to smoke.	83 (166)	3031	3.85	1.28	.05(0.32)	-0.85, 0.79
*Study 2*: *Physical Activity*							
1. Daily MVPA (minutes)		61 (120)	2731	48.92	27.55	.32(.32)	-.31, 1.00
2. Daily Action Control	Today I had my intentions in terms of my physical activity constantly on my mind *(awareness of standards)* Today I constantly monitored whether I acted the way I intended to in terms of my physical activity *(self-monitoring)* Today I did my best to be physically active the way I intended to *(self-regulatory effort)*	61 (120)	2660	3.23	0.98	.15(.26)	-.34, .82
3. Daily Intentions	I intend to be physically active tomorrow.	61 (120)	2660	3.84	1.09	.17(.28)	-.56, .88
4. Daily Self-Efficacy	I am confident that I can be physically active tomorrow even if it is difficult.	61 (120)	2660	3.69	1.10	.13(.36)	-1.00, .93
5. Daily Action Planning	For tomorrow, I planned exactly how I will be physically active.	61 (120)	2660	3.30	1.27	.13(.30)	-.39, .90

*Note*. *N* = number of couples (individuals), *n* = number of available diary days. We reported the means of the person-specific mean levels *(M)* and the average within-person standard deviation *(SD*_*w*_*)*. We also reported the means, standard deviation, minimum and maximum of the within-couple correlation *(R*_*W*_*)*.

^a^ Note that of two couples one partner had no data on the outcome, resulting in a total of 120 individuals in Study 2.

In Study 1, *daily number of cigarettes smoked* was assessed by the item “Did you smoke today (including only one puff)?” with the response format of no (0) and yes (1), and if yes, “How many cigarettes did you smoke today?” If participants had not smoked, number of cigarettes smoked was coded as 0. Non-integers were conservatively rounded to the next higher value.

In Study 2, *daily MVPA in minutes* was assessed with triaxial GT3X+ monitors (ActiGraph, Pensacola, FL) worn at the right hip during waking hours. For each participant, a sum per day was calculated for the total minutes of MVPA (>2690 cpm in vector magnitude; [30]). Only days with at least 10 hours of valid wear time were included in the analyses, with non-wear time filtered based on an algorithm of ≥ 90min of consecutive zeros in vector magnitude [31]. For more details on data processing see Berli et al. [28]. Across 61 participating couples included into the final sample, *n* = 2,731 [83.2%] out of 3,294 possible diary days were available and analyzed.

### Data analysis

Data from two independent samples of heterosexual couples were analyzed using the Actor-Partner Interdependence Model (APIM; [24]). Actor (the individual) and partner (the individual’s partner) scores of all volitional HAPA variables were used, allowing to estimate the extent to which the health behavior outcome is related to one’s own and one’s partner’s predictor scores while controlling simultaneously for the effect of both. As both studies assessed dyadic intensive longitudinal data, we employed multilevel modeling to account for the interdependence among couple members’ daily observations, using a two-level statistical model for distinguishable dyads as is indicated for heterosexual couples [32]. Each predictor variable was first decomposed into individual mean levels across the diary days (i.e., between-person variance) and the daily fluctuations around these means (i.e., within-person variance). The between-person predictor variables were grand-mean centered to allow for a meaningful interpretation of the intercept. We tested independent APIM models for each volitional HAPA predictor separately, as too many predictors may render a model less stable and less precise [33].

To investigate the effects of volitional self-regulatory strategies on same day and next day health behavior, prospective lagged models were applied: We modeled each couple member’s outcome on a given day *t* as a function of one’s own fluctuation in the respective volitional HAPA predictor and one’s partner’s fluctuation in the respective HAPA predictor the previous day (*t*-1) as well as that same day (*t)* (within-person actor and partner predictors), adjusting for the mean level of one’s own and one’s partner’s respective HAPA predictor (between-person actor and partner predictors). Additionally, all models were statistically adjusted for fluctuations in the outcome of the previous day (*t*-1) to capture change from the previous to the next day. Thus, for each of the four HAPA predictors the same set of models was run, but in reporting the results we focused on associations that aligned with the theory-based hypotheses: Because action control is conceptualized as a concurrent self-regulatory strategy, we focused on same day associations. Because intention, self-efficacy, and action planning are conceptualized as prospective self-regulatory strategies, we focused on next day associations. Complete statistical results are reported in the Supporting Information. In all models, we included gender as a covariate and a time variable representing the diary days (centered on the first available day) to model linear effects over time. In Study 1, data were available for all 22 days (the joint self-set quit date itself and 21 days after), because reports before the quit date were obtained. In Study 2 the first diary day was omitted from analyses, because predictor and outcome reports from the previous day were not available, resulting in 27 days. Because we did not have a priori hypotheses as to gender differences in the effects, we constrained actor and partner effects to be equal. Sensitivity analyses including interaction terms with gender for all within- and between-person predictors to allow for potential differences in effects between men and women (cf. Kenny et al., 2006) did not result in improved model fit. Thus, the more parsimonious models are reported below.

Finally, we specified a maximal random effects structure [34] including random intercept and slopes for all lower level predictors, using a variance components (VC) covariance structure. In case of non-convergence, the random effects structure was successively reduced until convergence was met. All analyses were run in SPSS 25. To predict the count variable *daily number of cigarettes smoked* (Study 1), we used a generalized linear mixed Poisson model with a logarithmic link function [35]. The effect sizes for Poisson models are rate ratios (*RR*). To predict the variable *daily MVPA in minutes (*Study 2), we used a linear mixed model and included hours of *device wear-time* per day (centered around the grand-mean) as a covariate to adjust for the potential impact of varying levels of wear-time. Additional sensitivity analyses revealed that results did not change when including age, relationship length, and weekend day as covariates, and therefore the more parsimonious models are reported.

## Results

### How concordant are couples’ volitional self-regulation processes in daily life?

To examine the concordance of couples’ volitional self-regulation in daily life, we calculated within-couple correlations for each HAPA predictor as well as the outcome, describing the extent to which one partner’s fluctuation in volitional self-regulation around their person-mean on a given day covaries with the other partner’s fluctuation in volitional self-regulation around their person-mean that same day. As shown in Table 1, the average correlation between female and male partners in the four volitional HAPA predictors was positive but small, ranging from .05 to .19 in Study 1, and from .13 to .17 in Study 2. However, the range of the within-couple correlations was high, indicating that some couples covaried highly and positively in their daily self-regulation over time, while others covaried in the opposite manner.

### Do one’s own volitional HAPA processes predict one’s own same day and next day health behavior (actor effects)?

In line with the HAPA, we hypothesized that daily action control would be associated with better health behavior (less cigarettes smoked, more MVPA) the same day, and that daily intention, self-efficacy and action planning reported for the next day would be associated with better health behavior the next day. Please note that separate APIM models were conducted for each predictor in order to facilitate model convergence and the specification of maximal random effects. Fixed effects on same day and next day health behavior are summarized in Table 2. For complete statistical results see S1 and S2 Tables.

**Table 2:** Fixed effects estimates from mixed models for same day and next day associations between volitional HAPA predictors and numbers of cigarettes smoked (Study 1), and minutes of moderate-to-vigorous physical activity (MVPA) (Study 2).

		Numbers of cigarettes smoked (Study 1)			Minutes of MVPA (Study 2)		
Fixed Effects	Estimate	*RR*	*LL CI*	*UL CI*	Estimate	*LL CI*	*UL CI*
Same Day Associations (*t)*							
Intercept	−0.27	0.77	0.51	1.14	48.38***	42.97	53.80
Gender	0.07	1.08	0.80	1.45	4.94	−2.15	12.02
Same day *Action Control* (actor effect)	−0.31***	0.73	0.67	0.80	8.73***	6.63	10.83
Same day partner’s *Action Control* (partner effect)	−0.11***	0.90	0.85	0.95	2.12**	0.60	3.63
Next Day Associations (*t*-1*)*							
Intercept	−0.06	0.94	0.68	1.31	49.48***	44.00	54.95
Gender	0.18	1.20	0.93	1.55	4.57	−2.81	11.96
Previous day *Intentions* (actor effect)	−0.17***	0.84	0.79	0.90	4.35***	3.03	5.67
Previous day partner’s *Intentions* (partner effect)	−0.04**	0.96	0.94	0.99	1.21^†^	−0.10	2.52
Intercept	−0.15	0.87	0.63	1.18	49.41***	43.84	54.97
Gender	0.11	1.11	0.86	1.45	4.66	−2.57	11.89
Previous day *Self-Efficacy* (actor effect)	−0.16***	0.85	0.79	0.91	4.89***	3.45	6.32
Previous day partner’s *Self-Efficacy* (partner effect)	−0.02^†^	0.98	0.95	1.00	0.67	−0.62	1.96
Intercept	−0.23	0.79	0.47	1.33	50.20***	44.68	55.71
Gender	0.06	1.06	0.78	1.44	5.91	−1.43	13.26
Previous day *Action Planning* (actor effect)	−0.08**	0.92	0.87	0.97	4.15***	2.89	5.41
Previous day partner’s *Action Planning* (partner effect)	0.01	1.01	0.95	1.07	0.56	−0.62	1.75

Note. *N* = 83 couples with a maximum of 22 days (*n* = 3026 available days) in Study 1, *N* = 61 couples with a maximum of 27 days (*n* = 2731 available days) in Study 2. *RR* = Rate Ratio, *LL CI* = Lower level 95% Confidence Interval, *UL CI* = Upper Level 95% Confidence Interval. Gender was coded: Female = -0.5 and Male = 0.5. Analyses were adjusted for time, time by gender, mean level of volitional HAPA predictor and partner’s mean level of volitional HAPA predictor, outcome on previous day, volitional HAPA predictor on previous day (in model testing same-day associations), volitional HAPA predictor on same day (in models testing next day associations) and device wear-time (only Study 2).

^†^p < .10

*p < .05

**p < .01

***p < .001

In Study 1, the average level of daily number of cigarettes smoked on day 0 (i.e., when all covariates were zero) was between 0.77 and 0.94 cigarettes, depending on the model. Men and women did not differ in their number of cigarettes smoked. As hypothesized, significant actor effects emerged for all volitional predictors of the HAPA. For the average participant, higher levels of one’s own *action control* on a given day (1-unit increase above person-specific mean) predicted a lower level of one’s own number of cigarettes smoked that same day (*p* < .001), while adjusting for the previous day’s level of action control and number of cigarettes smoked. The reduction in number of cigarettes smoked was 27% on days with higher action control than usual. Also, higher levels of one’s own *intention*, *self-efficacy*, and *action planning* on a given day (1-unit increase above person-specific mean) predicted a lower level of one’s own number of cigarettes smoked the next day (all *p* < .01), while adjusting for the previous day’s level of number of cigarettes smoked and the same day’s level of the respective volitional HAPA predictor. The reduction in number of cigarettes smoked was 16%, 15% and 8% on days with higher intention, self-efficacy, and action planning than usual, respectively.

The random effects revealed that there was considerable variation between individuals in their average level of number of cigarettes smoked (random intercept), and the extent to which one’s own same day action control and previous day intention, self-efficacy and action planning were associated with same day number of cigarettes smoked (random slopes for actor effects).

In Study 2, results showed that the average level of daily MVPA on day 0 (i.e. when all covariates were zero) was between 48.37 and 50.20 minutes, depending on the model. Men were not significantly more active than women. As hypothesized, significant actor effects emerged for all volitional predictors of the HAPA. For the average participant, higher levels of one’s own *action control* on a given day (1-unit increase above person-specific mean) predicted more minutes of one’s own MVPA that same day (*p* < .001), while adjusting for the previous day’s level of action control and MVPA. Also, higher levels of one’s own *intention*, *self-efficacy*, and *action planning* on a given day (1-unit increase above person-specific mean) predicted more minutes of one’s own MVPA the next day (all *p* < .001), while adjusting for the previous day’s level of MVPA and the same day’s level of the respective volitional HAPA predictor.

The random effects revealed that there was considerable variation between individuals in their average level of MVPA (random intercept), and the extent to which one’s own same day action control and previous day intention (albeit only marginally significant), self-efficacy and action planning were associated with same day MVPA (random slopes for actor effects).

### Do one’s partner’s volitional HAPA processes predict one’s own same day and next day health behavior (partner effects)?

We examined whether one’s partner’s daily action control, intentions, self-efficacy, and action planning would independently predict one’s own health behavior (less cigarettes smoked, more MVPA) that same day or the next day, respectively. Fixed effects on same day and next day health behavior are summarized in Table 2. For complete statistical results see S1 and S2 Tables.

In Study 1, partner effects were found for action control, intention, and self-efficacy for the average participant. Over and above the effect of one’s own *action control*, higher levels of one’s partner’s action control on a given day predicted a lower level of one’s own number of cigarettes smoked that same day (*p* < .001), while adjusting for the previous day’s level action control and number of cigarettes smoked. This effect amounts to a reduction of 10% in one’s own number of cigarettes smoked. Higher levels of one’s partner’s *intention* (*p* < .01) on a given day predicted a lower level of one’s own number of cigarettes smoked the next day, while adjusting for the previous day’s level of number of cigarettes smoked and the same day’s level of intention. The reduction in one’s own number of cigarettes smoked was 4%. For *self-efficacy* a marginal significant partner effect emerged (*p* = .058). Reduction in one’s own number of cigarettes smoked was 2%. We did not find any partner effect for *action planning*, indicating that one’s partner’s action planning on a given day was not associated with a lower level on one’s own number of cigarettes smoked the next day over and above the effects of one’s own level in this predictor.

The random effects indicated that there was considerable variation between individuals in the extent to which one’s partner’s same day action control and previous day action planning were associated with same day number of cigarettes smoked (random slopes for partner effects).

In Study 2, partner effects were found for action control and intention for the average participant. Over and above the effect of one’s own *action control*, higher levels of one’s partner’s action control on a given day predicted more minutes of one’s own MVPA that same day (*p* < .01), while adjusting for the previous day’s level of action control and MVPA. Higher levels of one’s partner’s *intention* on a given day tended to predict more minutes of one’s own MVPA the next day (*p* = 0.070), while adjusting for the previous day’s level of MVPA and the same day’s level of intention. We did not find any partner effects for *self-efficacy* and *action planning*, indicating that one’s partner’s self-efficacy and action planning on a given day was not associated with more minutes of one’s own MVPA the next day over and above the effects of one’s own level in these predictors.

The random effects showed that there was considerable variation between individuals in the extent to which one’s partner’s same day action control and previous day self-efficacy (albeit only marginally significant) and action planning were associated with same day minutes of MVPA (random slopes for partner effects).

## Discussion

This research contributes to the health behavior change literature by transferring the volitional phase of the HAPA to a dyadic level, and elucidating the effects of volitional processes for one’s own (actor effects) and one’s partner’s (partner effects) health behavior on a day-to-day basis. The patters of associations were illustrated with data obtained in two independent dyadic daily diary studies, focusing on volitional self-regulation processes within romantic couples aiming to quit smoking (Study 1) and to become physically active (Study 2).

Across the two studies, three main findings emerged. First, we found a substantial positive within-couple correlation in health behavior that is in line with previous evidence [18, 19]. Additionally, our results suggest that romantic partners covary positively to some degree, with high variability across couples. Thus, it seems that interdependence among romantic couples is not limited to their health behavior, but also to their effort in regulating it, as proposed by the TGD theory [20]. Future work should attempt to identify differences at the couple-level (e.g., relationship satisfaction) that can explain the variability in self-regulation interdependence among romantic couples. This may allow for progress towards personalized medicine approaches in behavior change science, clarifying for whom health behavior change interventions involving the partner may be relevant, and for whom not.

Second, findings consistently indicated that as hypothesized one’s own action control, as well as one’s own intentions, self-efficacy and planning are associated with better own health behavior (less cigarette smoking and higher MVPA) that same day or the next day, respectively. These actor effects are in line with findings from previous studies (e.g., [9, 10, 11] and confirm the validity of the volitional phase of the HAPA model from a within-person perspective in the context of smoking cessation and physical activity.

Third, we tested for spillover effects between partners, that is whether one’s partner’s volitional self-regulation process would independently contribute to one’s own health behavior. Examining not only actor effects but also partner effects in health behavior change has barely received attention. Across both studies, we found that partner’s action control and intention (albeit only marginally significant in the context of physical activity) contributed to better own health behavior (less cigarettes smoked and more MVPA) that same or the next day. Also consistently across both studies, we did not find any partner effect for individual action planning. Howland et al. [22] previously found that partner’s higher perceived behavioral control predicted higher own intentions to be physically active. However, this finding was not consistently replicated in the present research. Partner’s self-efficacy was only in tendency associated with less cigarettes smoked (Study 1), but not more minutes of MVPA (Study 2).

Taken together, the present results provide novel evidence that individual regulation processes, action control and intention in particular, are not constrained to the individual in terms of their effects on behavior, but can be of dyadic relevance in close relationships. Relatedly, this suggests that the interdependency in health behavior is not entirely driven by interdependent self-regulation processes operating via actor effects, but a mixture of actor and partner effects (i.e., couple-level pattern). This might be particularly relevant in the context of dyads that have the possibility to jointly initiate and maintain health behavior change. However, studies are needed that replicate the present findings on partner effects.

The findings pose the question about potential mechanisms underlying such a couple-level pattern. One possibility is that couples develop and engage in dyadic forms of self-regulation such as formulating joint intentions, monitoring each other’s progress (i.e., dyadic action control; [28]), or being confident that together they can engage in behavior change efforts (i.e., dyadic efficacy; [36]). Similarly, the concept of dyadic planning [37] or collaborative planning [38] has been introduced. Our findings showed no partner effects for individual action planning. It may be that individual action plans are often highly specific to the individual’s own daily schedules etc., and may thus not necessarily benefit the partner. Partner effects in terms of planning might be more obvious in asymmetric dyads such as a spouse caring for a patient [37]. It is also possible that accounting for dyadic planning instead of individual action planning may uncover partner effects of planning variables.

It needs to be highlighted that the partner effects reported concern average effects across the sample. Similar to the high variability in within-couple concordance of volitional self-regulation, it needs to be assumed that partner effects are not the equal across couples. We allowed for random variance in partner effects across partners, but variance was too small to systematically investigate patterns of heterogeneity. However, future research should address such issues with samples large enough to test this.

### Strengths and limitations

The present article has several strengths. In both studies we collected intensive longitudinal data with daily diaries allowing to understand life as it is lived, and within individuals and couples. Moreover, we applied a dyadic perspective by examining independent reports from both partners of heterosexual romantic couples and applied a theoretical backdrop of the HAPA model to elucidate self-regulation in a dyadic context. This enabled us to study potential couple-level influence of individual self-regulation processes in health behavior change by investigating spillover effects from one’s partner’s self-regulation on one’s own health behavior. Furthermore, we established a dyadic level of the HAPA across two different health behaviors (i.e., quitting smoking, increasing physical activity). An additional strength of Study 2 lies in the objective assessment of MVPA via accelerometers. In Study 1 no objective measure of smoking abstinence at the daily level was employed. However, both partners’ reports of smoking abstinence was biochemically verified with a carbon monoxide test of expelled air [26]: All participants reporting continuous abstinence one month after the joint quit date were detected as non-smokers by the objective point-prevalence measure. Furthermore, both studies used prospective lagged analyses to account for temporal order in the effects. As hypothesized, the prospective self-regulation strategies intention, self-efficacy and action planning yielded prospective associations with next day behavior whereas the concurrent strategy action control yielded same-day associations. Results did not differ when including age, relationship length, and weekend day as covariates.

Despite this, no causality can be established. This presents a limitation, particularly for the concurrent associations between action control and same day health behavior. To establish causality, an experimental design would be needed. Previous studies tested spillover effects of self-regulation intervention in couples [39]. A further limitation is that in both studies the volitional HAPA predictors were assessed with single items to keep the daily diaries short and participants’ burden low. However, there is evidence that the single-items may serve as valid and useful measures [40]. The studies could consistently show associations as hypothesized, strengthening their validity. Moreover, action control was assessed with three items in Study 2 (one each for the three subfacets) as opposed to one item in Study 1 (only one item for self-monitoring), but results were highly comparable. Nevertheless, an in depth validation of the single items seems advisable. Another limitation is that recruiting community members via advertisements may have entailed a bias due to self-selection. The present study focused on romantic couples with relatively strict inclusion criteria, and results may thus not generalize to other samples or dyadic constellations. Examining other forms of close relationships such as best friend dyads or parent-child dyads may be informative as to the role of the wider social context in health behavior change.

### Conclusions

The present research highlights the importance of individual regulation processes at a dyadic level, which has vital implication for theory development and clinical practice. Health promotion programs should account for the interdependency of individuals with close relationship partners. So far, guidelines on smoking cessation emphasize the importance of the social environment in the context of health behavior change [41]. Our findings point towards specific dyadic processes that may be related to behavior change. For example, our results suggest that a useful approach for health behavior modification may be to recruit couples to jointly initiate and maintain behavior change, because individual’s self-regulation effort may provide additional benefits for the partner’s behavior change.

In sum, the present research demonstrates the applicability of transferring the HAPA model to a dyadic level of romantic couples facing a joint health behavior change. Previous findings on associations between individual’s volitional self-regulation processes and health behavior change in daily life (actor effects) were replicated in a dyadic context. Furthermore, the present studies provide first insights that spillover effects from one partner’s self-regulation to the other partner’s health behavior (partner effects) are possible in daily life. Adding the couple-level processes may enable us to better understand health behavior change processes and to develop more effective interventions.

## Supporting information

S1 Table Parameter estimates from mixed poisson models testing the within-person effects of daily volitional HAPA predictors on daily number of cigarettes smoked in the context of smoking cessation (Study 1). (DOCX)

S2 Table Parameter estimates from mixed models testing the within-person effects of daily volitional HAPA predictors on daily physical activity in the context of physical activity (Study 2). (DOCX)
