# Supplementary material for: Couples' daily self-regulation: The Health Action Process Approach at the dyadic level
Source: PLoS One. 2018 Oct 29;13(10):e0205887. doi: 10.1371/journal.pone.0205887 (PMC6205589; doi:10.1371/journal.pone.0205887)
Supplement: S1 Table — (DOCX) [file pone.0205887.s001.docx]

|  | *Action control* | | |  | *Intention* | | |  | *Self-efficacy* | | |  | *Action planning* | | |
| --- | --- | --- | --- | --- | --- | --- | --- | --- | --- | --- | --- | --- | --- | --- | --- |
| Fixed effects | Estimate | *SE* | *RR* |  | Estimate | *SE* | *RR* |  | Estimate | *SE* | *RR* |  | Estimate | *SE* | *RR* |
| Intercept | −0.27 | 0.20 | 0.77 |  | −0.06 | 0.17 | 0.94 |  | −0.15 | 0.16 | 0.87 |  | −0.23 | 0.26 | 0.79 |
| Gender | 0.07 | 0.15 | 1.08 |  | 0.18 | 0.13 | 1.20 |  | 0.11 | 0.13 | 1.11 |  | 0.06 | 0.16 | 1.06 |
| Time | −0.01 | 0.01 | 0.99 |  | −0.01 | 0.01 | 0.99 |  | −0.01 | 0.01 | 0.99 |  | −0.002 | 0.01 | 1.00 |
| Gender x Time | 0.01 | 0.004 | 1.01 |  | 0.01* | 0.004 | 1.01 |  | 0.01** | 0.004 | 1.01 |  | 0.01^†^ | 0.004 | 1.01 |
| Mean volitional HAPA predictor | −0.99*** | 0.12 | 0.37 |  | −1.31*** | 0.11 | 0.27 |  | −1.20*** | 0.10 | 0.30 |  | −0.33* | 0.13 | 0.72 |
| Partner’s mean volitional HAPA predictor | −0.54*** | 0.12 | 0.59 |  | −0.55*** | 0.11 | 0.58 |  | −0.54*** | 0.10 | 0.59 |  | 0.05 | 0.13 | 1.06 |
| Previous day outcome | 0.01 | 0.01 | 1.01 |  | 0.01 | 0.01 | 1.01 |  | 0.01 | 0.01 | 1.01 |  | 0.01 | 0.01 | 1.01 |
| Previous day volitional HAPA predictor | −0.04** | 0.01 | 0.96 |  | −0.17*** | 0.03 | 0.84 |  | −0.16*** | 0.04 | 0.85 |  | −0.08** | 0.03 | 0.92 |
| Previous day partner’s volitional HAPA predictor | 0.01 | 0.01 | 1.01 |  | −0.04** | 0.01 | 0.96 |  | −0.02^†^ | 0.01 | 0.98 |  | 0.01 | 0.03 | 1.01 |
| Same day volitional HAPA predictor | −0.31*** | 0.05 | 0.73 |  | −0.11** | 0.03 | 0.90 |  | −0.15*** | 0.04 | 0.86 |  | −0.11** | 0.04 | 0.90 |
| Same day partner’s volitional HAPA predictor | −0.11*** | 0.03 | 0.90 |  | −0.02 | 0.01 | 0.98 |  | −0.03^†^ | 0.01 | 0.97 |  | −0.03 | 0.02 | 0.97 |
|  |  |  |  |  |  |  |  |  |  |  |  |  |  |  |  |
| Random effects ([co-]variances)^a^ |  |  |  |  |  |  |  |  |  |  |  |  |  |  |  |
| Level 2 (between-person) |  |  |  |  |  |  |  |  |  |  |  |  |  |  |  |
| Intercept | 2.96*** | 0.59 |  |  | 1.88*** | 0.37 |  |  | 1.70*** | 0.34 |  |  | 5.30*** | 1.00 |  |
| Gender | 1.30*** | 0.29 |  |  | 0.86*** | 0.21 |  |  | 0.95*** | 0.22 |  |  | 1.33*** | 0.30 |  |
| Time | 0.01** | 0.001 |  |  | 0.01** | 0.001 |  |  | 0.01*** | 0.001 |  |  | 0.01** | 0.001 |  |
| Previous day outcome | 0.003** | 0.001 |  |  | 0.002* | 0.001 |  |  | 0.002* | 0.001 |  |  | 0.004** | 0.001 |  |
| Previous day volitional HAPA predictor | 0.002 | 0.001 |  |  | 0.04** | 0.01 |  |  | 0.05* | 0.02 |  |  | 0.03** | 0.01 |  |
| Previous day partner’s volitional HAPA predictor | - | - |  |  | - | - |  |  | - | - |  |  | 0.04* | 0.02 |  |
| Same day volitional HAPA predictor | 0.13*** | 0.03 |  |  | 0.03* | 0.01 |  |  | 0.07^†^ | 0.02 |  |  | 0.06** | 0.02 |  |
| Same day partner’s volitional HAPA predictor | 0.03** | 0.01 |  |  | 0.001 | 0.001 |  |  | 0.001 | 0.001 |  |  | 0.01 | 0.01 |  |
| Level 1 (within-person) |  |  |  |  |  |  |  |  |  |  |  |  |  |  |  |
| Residual | 0.59*** | 0.03 |  |  | 0.85*** | 0.03 |  |  | 0.81*** | 0.03 |  |  | 0.74*** | 0.03 |  |
| Autocorrelation | 0.32*** | 0.04 |  |  | 0.19*** | 0.03 |  |  | 0.17*** | 0.03 |  |  | 0.19*** | 0.03 |  |

S1 Table *Parameter estimates from mixed poisson models testing the within-person effects of daily volitional HAPA predictors on daily number of cigarettes smoked in the context of smoking cessation (Study 1)*

Note. *N* = 83 couples with a maximum of 22 days, *n* = 3026 available days. *SE =* standard error, *RR* = rate ratio. Gender was coded: Female = -0.5 and Male = 0.5. ^a^Due to non-convergence, some of the random effects could not be computed. ^†^p < .10, *p < .05, **p < .01, ***p < .001.
