# Supplementary material for: Couples' daily self-regulation: The Health Action Process Approach at the dyadic level
Source: PLoS One. 2018 Oct 29;13(10):e0205887. doi: 10.1371/journal.pone.0205887 (PMC6205589; doi:10.1371/journal.pone.0205887)
Supplement: S2 Table — (DOCX) [file pone.0205887.s002.docx]

S2 Table *Parameter estimates from mixed models testing the within-person effects of daily volitional HAPA predictors on daily physical activity in the context of physical activity (Study 2)*

|  | *Action control* | |  | *Intention* | |  | *Self-efficacy* | |  | *Action planning* | |
| --- | --- | --- | --- | --- | --- | --- | --- | --- | --- | --- | --- |
| Fixed effects | Estimate | *SE* |  | Estimate | *SE* |  | Estimate | *SE* |  | Estimate | *SE* |
| Intercept | 48.38*** | 2.72 |  | 49.48*** | 2.75 |  | 49.41*** | 2.79 |  | 50.20*** | 2.77 |
| Gender | 4.94 | 3.58 |  | 4.57 | 3.73 |  | 4.66 | 3.65 |  | 5.91 | 3.71 |
| Time | 0.02 | 0.10 |  | −0.07 | 0.10 |  | −0.06 | 0.10 |  | −0.13 | 0.09 |
| Gender x Time | 0.24 | 0.17 |  | 0.28 | 0.18 |  | 0.28 | 0.18 |  | 0.27 | 0.18 |
| Mean volitional HAPA predictor | 2.20 | 2.45 |  | 0.12 | 2.41 |  | 2.21 | 2.46 |  | 2.73 | 2.37 |
| Partner’s mean volitional HAPA predictor | −3.30 | 2.48 |  | −3.52 | 2.38 |  | −2.79 | 2.49 |  | −3.99^†^ | 2.39 |
| Device wear-time (in hours) | 2.04*** | 0.44 |  | 2.10*** | 0.50 |  | 2.20*** | 0.49 |  | 2.13*** | 0.49 |
| Previous day outcome | −0.11*** | 0.03 |  | −0.12*** | 0.03 |  | −0.12*** | 0.03 |  | −0.12*** | 0.03 |
| Previous day volitional HAPA predictor | 1.09^†^ | 0.62 |  | 4.35*** | 0.66 |  | 4.89*** | 0.71 |  | 4.15*** | 0.63 |
| Previous day partner’s volitional HAPA predictor | 0.05 | 0.54 |  | 1.21^†^ | 0.65 |  | 0.67 | 0.64 |  | 0.56 | 0.59 |
| Same day volitional HAPA predictor | 8.73*** | 1.05 |  | −0.67 | 0.53 |  | −0.43 | 0.50 |  | −0.16 | 0.44 |
| Same day partner’s volitional HAPA predictor | 2.12** | 0.75 |  | −0.22 | 0.50 |  | −0.20 | 0.50 |  | 0.14 | 0.44 |
|  |  |  |  |  |  |  |  |  |  |  |  |
| Random effects ([co-]variances)^a^ |  |  |  |  |  |  |  |  |  |  |  |
| Level 2 (between-person) |  |  |  |  |  |  |  |  |  |  |  |
| Intercept | 349.83*** | 72.81 |  | 350.47*** | 73.03 |  | 367.23*** | 75.53 |  | 362.41*** | 74.72 |
| Gender | 403.21*** | 96.47 |  | 411.78*** | 101.00 |  | 404.52*** | 99.25 |  | 389.44*** | 95.96 |
| Time | 0.12 | 0.09 |  | 0.05 | 0.08 |  | 0.03 | 0.08 |  | 0.04 | 0.08 |
| Device wear-time (in hours) | 5.61** | 2.05 |  | 8.13** | 2.72 |  | 7.25** | 2.55 |  | 7.25** | 2.52 |
| Previous day outcome | 0.01** | 0.01 |  | 0.02** | 0.01 |  | 0.02** | 0.01 |  | 0.01** | 0.01 |
| Previous day volitional HAPA predictor | 1.91 | 3.62 |  | 7.38^†^ | 4.22 |  | 11.60* | 5.43 |  | 9.82* | 4.32 |
| Previous day partner’s volitional HAPA predictor | - | - |  | 7.53^†^ | 4.10 |  | 7.18^†^ | 4.09 |  | 7.70* | 3.69 |
| Same day volitional HAPA predictor | 43.18*** | 11.84 |  | 0.77 | 2.71 |  | - | - |  | - | - |
| Same day partner’s volitional HAPA predictor | 13.54* | 6.71 |  | - | - |  | - | - |  | - | - |
| Level 1 (within-person) |  |  |  |  |  |  |  |  |  |  |  |
| Residual | 711.25*** | 26.05 |  | 807.45*** | 29.47 |  | 797.35*** | 28.52 |  | 795.23*** | 28.50 |
| Autocorrelation | 0.21*** | 0.04 |  | 0.20*** | 0.04 |  | 0.19*** | 0.04 |  | 0.20*** | 0.04 |

Note. *N* = 61 couples with a maximum of 27 days (*n* = 2731 available days). *SE =* standard error. Gender was coded: Female = -0.5 and Male = 0.5. ^a^Due to non-convergence, some of the random effects could not be computed. ^†^p < .10, *p < .05, **p < .01, ***p < .001.
